# Youth report of healthcare transition counseling and autonomy support from their rheumatologist

**DOI:** 10.1186/1546-0096-10-36

**Published:** 2012-11-14

**Authors:** Courtney Kellerman Wells, Barbara J McMorris, Keith J Horvath, Ann W Garwick, Peter B Scal

**Affiliations:** 1 University of Minnesota, School of Social Work, Minneapolis, MN, USA; 2 University of Minnesota, School of Nursing, Minneapolis, MN, USA; 3 University of Minnesota, School of Public Health, Minneapolis, MN, USA; 4 University of Minnesota, School of Medicine, Minneapolis, MN, USA; 5 Department of Pediatrics, University of Minnesota, 717 Delaware St. SE, Room 359, Minneapolis, MN, 55455, USA

**Keywords:** Healthcare transition, Autonomy support, Adolescent health, Young adult, Arthritis, Counseling, Chronic condition

## Abstract

**Background:**

To increase understanding of the healthcare transition (HCT) process for young people living with Juvenile Idopathic Arthritis (JIA) by examining: 1) the extent to which youth report discussing HCT topics with their rheumatologist and 2) the association between youth perceptions of autonomy support from their rheumatologist and HCT discussions.

**Methods:**

Data are from an online survey of youth in the United States with rheumatologic conditions (n= 134). HCT discussion was measured by 4 questions from the *National Survey of Children with Special Health Care Needs.* Youth perception of autonomy support was measured using a validated 6-item scale*.*

**Results:**

One third of the youth (33.7%) reported talking to their rheumatologist about transferring to adult medicine. Less than half (40.8%) of respondents talked with their rheumatologist about adult healthcare needs, and less than a quarter (22.0%) discussed acquiring health insurance as an adult. Nearly two-thirds of respondents (62.7%) reported that their rheumatologist usually/always encourages self-care responsibility. Multivariate analyses revealed significant associations between rheumatologist support for youth autonomy and HCT counseling.

**Conclusion:**

The low frequency of HCT counseling reported indicates a continuing need to increase awareness among rheumatologist in the USA. The strong associations between rheumatologist’s support for youth autonomy and HCT counseling suggest that developmentally “in-tune” providers may deliver the best guidance about transition planning for youth living with arthritis.

## Background

Advances in the treatments of many chronic conditions of childhood offer the promise of improved quality of life and a long and productive adulthood. In rheumatology, the biologic disease-modifying antirheumatic drugs have resulted in marked reductions in disease activity and medication side effects for adolescents growing up with rheumatologic conditions like Juvenile Idiopathic Arthritis (JIA) [1]. Despite these advances, no cure exists for these conditions and most adolescents can expect to experience adulthood with the need for intensive and expensive medical care [2-4]. Preparing youth and their families for the transition to adulthood and from pediatric to adult healthcare, called “healthcare transition” (HCT), through the support of healthcare providers and systems is an international priority [2,5]. Changing systems and practice has proven to be difficult. The most recent US data demonstrate that little progress has been made over the past decade increasing the proportion of youth who have received HCT counseling from their providers; as of 2009 fewer than half of youth with special health care needs are receiving [6].

HCT counseling, guiding youth through the process of engaging in their healthcare self-management, is a recommended component of the adolescent health visit [7,8]. But evidence on effective counseling strategies is lacking [9-11]. Best practice in pediatrics suggests adopting a family centered approach focusing on anticipatory guidance and medical care counseling. Providers who use a family centered approach are more likely to engage youth and families in HCT counseling [12]. But attention to the developmental tasks of adolescence, individualization and autonomy, would suggest that counseling should, over time, support youth autonomy in self-management decision making and actions. Furthermore, autonomy support may be an important element of HCT counseling as it is identified as a central element of behavior change counseling and effective health behavior interventions [13,14].

Within the context of parent report of low rates of HCT counseling [15] and a lack of evidence on effective strategies, we set out to inform practice and program development by: 1) assessing the extent to which youth with JA themselves report having been counseled by their healthcare provider on HCT; and 2) test the association between youth perception of autonomy support from their healthcare providers and the provision of HCT counseling.

## Methods

### Data and sample

We conducted a needs assessment survey, MyRheum, to assess the health, healthcare needs and quality of life of US youth (age 14–19) with rheumatologic conditions (*n*= 134) between June and December 2010. Our questionnaire was constructed by selecting and adapting (where necessary) items from frequently used questionnaires and standardized measures. The survey included questions about general and arthritis-related health, healthcare use, quality and satisfaction, in addition to other topics relevant to youth growing up with JA. Since no population based list of youth with rheumatologic conditions exists from which to draw a representative sample, and recruiting a sample of youth through clinics would likely introduce bias as we evaluated the unmet needs, we recruited a community based sample using multiple recruitment strategies. In addition to fliers and letters distributed to youth seen in 5 pediatric rheumatology clinics in the upper Midwest we distributed flyers through relevant community based organizations, and made postings to social media websites such as Facebook and sites with youth related and rheumatologic related content. Potential participants were directed to the study website where they could review study information, self-report eligibility criteria (age 14–19 years, have arthritis, and live in the US) and complete an online consent process. The Institutional Review Board at the University of Minnesota approved consent procedures, including a waiver of parental consent and a restriction to youth age 14 years and older. Only young people who met the eligibility criteria and completed the consent process could access the online questionnaire. On average, respondents completed the 66-item survey in 22 minutes (ranging from 7–84 minutes). Youth who completed the questionnaire were mailed a $10 gift card. Each case was reviewed for extensive missing data, inconsistencies or markers for potential ineligible participation. This review resulted in the elimination of 5 response sets.

### Measures

Four questions, derived from the National Survey of Children with Special Health Care Needs asked specifically about HCT counseling [16]. These four questions are the dependent variables in this analysis. Respondents who reported seeing a pediatric rheumatologist were asked if they had discussed transferring care to a provider who treats adults. Then, all respondents were then asked whether they had discussed the following two questions with their rheumatologist: 1) how their healthcare needs in adulthood would change, and 2) about obtaining health insurance as an adult. Response options for these three questions were “yes” or “no.” Finally, participants were asked two transition related questions; 1)how often their rheumatologist or other provider encouraged them to take responsibility for their healthcare needs (e.g., taking medications, understanding their diagnosis, or following medical advice), with response options of “never,” “sometimes,” “usually,” and “always.” For ease of interpretation, responses were dichotomized into high (usually/always) and low (sometimes/never) levels of *self-care responsibility* and 2) whether, in the past 12 months, they had the chance to speak with their rheumatologist privately (“yes” or “no”).

The primary explanatory variable was participants’ perception of their rheumatologist’s support for autonomous medical care management. Autonomy support was measured by the 6-item version of the Healthcare Climate Questionnaire [17]. A sample item is: “I feel that my physician has provided me choices and options.” Participants responded to the items on a 7-point Likert scale ranging from 1 (*strongly disagree*) to 7 (*strongly agree*) and an overall score was calculated as the mean of the 6 items (Cronbach’s alpha = 0.95 in our sample). For ease of interpretation autonomy support was dichotomized using a median split into high (score that was at or above the median) and low levels (below the median).

Demographic variables included age (dichotomized as 14–16 and 17–19) to represent middle and late adolescence, sex, race/ethnicity, urban vs. rural residence (defined by postal code), and the number of parents or guardians in the household. The survey also asked participants to report their diagnoses (selecting more than one response was permitted from a list that included common and medical language to describe rheumatologic conditions associated with arthritis) and medications (check all that apply). The Pediatric Quality of Life Inventory [18] (PedsQL™: 15 items, Cronbach’s alpha = 0.90) was used to assess disease impact and yielded scores ranging from 0–100. Scores on the PedsQL™ were also dichotomized using a median split.

### Statistical analysis

We conducted univariate and bivariate analyses to describe sample characteristics and to examine group differences. We used logistic regression models to assess whether the association between autonomy support and each outcome noted in the bivariate analysis remained statistically significant and of the same directionand magnitude after adjusting for the potential cofounding of demographic characteristics. These regression models controlled for age, sex, race/ethnicity, residence, and PedsQL™ score. Analyses were conducted in PASW Statistics version 18 (IBM SPSS, Chicago, Illinois).

## Results

The sample was predominantly younger (age 14–16, 59.7%), female (83.6%), White (87.3%), and lived in a two-parent household (79.1%) (Table 1). Participants were from 21 states and most lived in urban areas (91.8%). The most frequently reported diagnosis was Juvenile Rheumatoid Arthritis/Juvenile Idiopathic Arthritis (JRA/JIA). The median score on the PedsQL™ was 58.3 (which is substantially lower than the mean of 83 among healthy youth [18]). The median autonomy support score was 5.67 (range 1 – 7, Std. Dev. 1.43) and weighted towards the high levels of autonomy support (skewness value of −0.90).

**Table 1:** Sample Characteristics (n=134)

	**%**
**Age, years (median, 16)**	
14 - 16	59.7
17 - 19	40.3
**Female**	83.6
**Race/ethnicity** (check all)	
White or European American	87.3
Hispanic or Latino	10.4
Black, African, or African American	4.5
American Indian or Native American	3.0
Asian, Asian American or Pacific Islander	0.7
Other	0.7
**Urban**	91.8
**Household composition**	
2 parent	79.1
1 parent	18.7
Other adult guardian	0.7
No parent or guardian	1.5
Reported diagnoses* (check all)	
JIA or JRA	66.4
Spondyloarthropathy	3.7
Systemic Lupus Erythematosus	3.0
Chronic recurrent multifocal osteomyelitis	1.5
Dermatomyositis	1.5
Other	23.9
**Current medication use** (check all)	
No medications	5.2
Nonsteroidal anti-inflammatory drugs	56.0
Disease-modifying anti-rheumatic drugs	64.2
Biologics	53.0
Other	31.3

* The five most frequently reported diagnoses.

Frequencies and percentages of transition-related counseling for the overall sample and by demographic group membership are shown in Table 2. Overall, one third (33.7%) of youth reported talking to their rheumatologist about transferring to adult medicine. Less than half (40.8%) of respondents had talked with a rheumatologist about their healthcare needs as they transitioned to adulthood, and less than a quarter (22.0%) discussed acquiring health insurance as an adult. Nearly two-thirds (62.7%) of respondents reported that their rheumatologist or healthcare providers usually or always encourage self-care responsibility. A larger proportion of older adolescents reported having a chance to see their rheumatologist privately (21 of 50; 41%) than did the younger adolescents (18 of 73, 25%) (p<.05). In bivariate analyses (Table 2), older youth reported having discussions about eventually seeing providers who treat adults and having discussions about the healthcare needs of adults more frequently than did younger youth. Youth report of a high level of autonomy support was not significantly associated with having discussions of insurance coverage or transfer to adult providers; however high level of autonomy support was associated with both discussing healthcare needs in adulthood and encouraging self-responsibility.

**Table 2:** Transition counseling by youth characteristics and perception of autonomy support

	**Discussed seeing providers who treat adults (% yes)**	**Discussed healthcare needs of adults (% yes)**	**Discussed acquiring insurance as an adult (% yes)**	**Providers encourage self-care responsibility (% usually or always)**
**Overall**	33.7	40.8	22.0	62.7
**Age**				
14-16	22.2	32.9	16.7	59.7
17-19	51.2*	51.9*	29.6	70.4
**Sex**				
Female	35.2	39.8	23.6	66.1
Male	25.0	45.6	13.6	54.6
**Race/ethnicity**				
White	50.0	40.0	22.4	62.6
Non-white	30.7	45.8	20.8	69.6
**Location**				
Urban	34.7	41.3	22.8	66.9
Rural	16.7	33.3	11.1	30.0*
**PedsQL (total)**				
Low	39.6	41.9	26.6	63.1
High	28.6	39.7	17.7	65.2
**Autonomy Support**				
Low	27.6	24.6	23.0	49.2
High	38.6	55.1*	21.1	77.1*

* Comparisons of independent variable values for each HCT question significant at *p* ≤ 0.05 by Pearson’s chi-square test.

Provider support for autonomy remained positively associated with discussing healthcare needs in adulthood and encouraging self-responsibility in the logistic regression models, controlling for age, sex, race/ethnicity, urban/rural residence, and PedsQL™ score. Specifically, youth who reported high level of autonomy support were significantly more likely to report that discussions of the healthcare needs of adults occurred (AOR = 3.89, 95% CI = 1.75-8.69) and that their providers usually or always encouraged self-care responsibility (AOR = 3.97, 95% CI = 1.74-9.05) compared to youth reporting low level of autonomy-support. And the relationship between autonomy support and the outcomes of having discussions of insurance coverage or transfer to adult providers was not statistically significant in regression models.

## Discussion and Conclusions

This survey provides a unique opportunity to examine HCT services from the perspective of youth themselves. There are two main findings from this study. First, based on youth report, we found low rates of discussing HCT issues by rheumatologists. Fewer than 4 in 10 youth recalled discussing health changes in adulthood, transfer to adult clinical care or maintaining health insurance coverage. Somewhat more encouraging, however, was that 6 in 10 youth reported that healthcare providers usually or always encouraged youth responsibility for healthcare. The validity of these findings is supported by the fact that they are remarkably consistent with reports from parents of children with arthritis [15]. These results, together with those of other studies, suggest that the majority of youth living with arthritis are not discussing HCT even though it is considered best practice. The second key finding was that youth perceptions of autonomy support from their healthcare providers was associated with higher likelihood of discussing self-responsibility and discussing the changing healthcare needs of adults. Autonomy is an important psychosocial developmental task of adolescence [19]. And autonomy support is increasingly recognized as an influential element of chronic care management. It appears that providers who youth perceive to be addressing this psychosocial task are the providers who are also providing developmentally appropriate healthcare services. Much of the HCT literature has advocated for clinical interventions that teach skills to youth living with chronic medical conditions, such as making appointments, reordering medications, and arranging the transfer of care to an adult service [2]. But it may be that the strategy of asking providers to add more and specific health education to the long list of anticipatory guidance and health promotion tasks may be ineffective. Instead, a strategy that encourages providers to take a developmentally targeted approach to clinical work with youth health may result in more and perhaps more youth centered approaches to preparing for adulthood. Our finding of a strong association between provider support for autonomy and HCT discussions suggests that developmentally “in-tune” providers may be the best prepared to provide guidance about transition planning for youth living with JA.

Supporting autonomy in healthcare settings refers to the processes of care that enhance a person’s sense of personal choice of therapeutic plans and personal behaviors [14]. Autonomy support is a key aspect of both patient centered care and adolescent development, and a key to successful healthcare transition. Healthcare providers can be autonomy supportive by seek to understand each adolescent’s experience of growing up with JIA, and involve them in identifying problems and resources, goals and strategies. The adolescent health interview strategy, HEADS [20], is one strategy that providers can use to become more patient centered and autonomy supportive. HEADS directs the adolescent health interview to areas of development and behavior that impact the current and future health of adolescents and which are likely to change over the transition to adulthood. HEADS topics include, but are not limited to Home, Education/Employment, Activities, Drugs, Depression, Sexuality, Safety. Conducting an interview in the HEADS order allows for exploration of topics in what are typically progressively more challenging topics. HEADS becomes a powerful autonomy support tool when the questions are used to trigger discussions of situations where an adolescent has successfully navigated a challenge, to elicit adolescent generated solutions and opportunities, and to model decision making. For example, the ‘home’ questions, “Tell me about your home and who you live with” become a patient centered, autonomy supportive transition intervention when they are expanded to include “How does having arthritis impact things around home?” or “How do you think that will change when you graduate high school?” or “Do you think having arthritis will affect your where you live when you go to college.” Autonomy support is enhance too, when adolescents have an opportunity to meet privately with their health care providers, and when they are assured confidentiality. Autonomy support in clinical settings with adolescents, especially those with chronic conditions, is challenging given the time constraints of visits and the focus on acute exacerbations and chronic management. Yet, the long term payoff from a program of autonomy support may be more efficient, effective and enjoyable patient communication and ultimately the successful transition to self-management and adult focused care.

The small sample size, sampling strategy and lack of sample heterogeneity limit the generalizability of our findings to the entire population of youth with arthritis. Our sample is primarily female, white and from two parent households and they all had access to a computer and the internet. It is likely that our sample represents, in general, a group with better access to resources than other youth with rheumatologic conditions. Thus we expect that our results may present a “best case” scenario in terms of the extent of healthcare transition counseling. Those youth with fewer resources may not be receiving as much in the way of transition services. In addition, results are derived from youth self-report and are not corroborated with medical records or provider reports. Thus, rates of counseling are subject to recall bias, and autonomy support is a perception of youth rather than an observed practice.

Further research on strategies for supporting young people during the process of HCT is necessary. Evidence to date has documented a strong need for HCT services, but there is little examination of the necessary components for effective programs [21]. Our findings highlight the importance of supporting the development of autonomy in young people living with JA and suggest that support for autonomy is related to the HCT process. Future research should identify key markers of autonomy-supportiveness in clinical interactions between young people with JA and their providers as well as examine whether support for autonomy predicts successful HCT in youth who are at the stage where this transition is appropriate.

Study findings also point to an opportunity for providers and educators to focus on training current and future rheumatology professionals (and others) to be expert in delivering developmentally targeted, youth-centered care. Finally, training providers to engage in clinical interactions that cultivate autonomy may equip youth with the skills necessary to take control of their disease management and prepare for the transfer of care to adult providers.

### Key message

Youth in the US report infrequent healthcare transition counseling from their rheumatologist.

Rheumatologists’ support for healthcare autonomy in young people is associated with HCT counseling

Improving clinician’s attention to the developmentally relevant autonomy supportive counseling may improve HCT counseling

## Competing interests

The authors have no conflicts of interests to report.

## Authors’ contribution

CW contributed to the conceptualization of the research question and methods, preliminary data analysis, interpretation of results and completed the first draft of the manuscript. BM contributed to the overall project design, data analysis plan and the integrity of the analysis, interpretation of results and manuscript preparation, AG and KH contributed to the overall project design, interpretation of results and manuscript preparation; PS participated in all aspects of the project including conceptualization of research questions, study design, data analysis, interpretation of results and completion of the manuscript subsequent to the first draft.

## Funding statement

This work was supported by the United States Health Resources and Services Administration, Maternal and Child Health Bureau through the Leadership Education in Adolescent Health Fellowship Training Program, [T71-MC-00006] to CKW and BJM); Agency for Healthcare Research and Quality [K08-HS015511, to PBS] and from the University of Minnesota-Academic Health Center Faculty Research Development Program [to PBS, AWG, KJH]

This work reflects the opinions of the authors and does not represent the opinions or influences of any of the funding agencies.
